# Seismic signature of the Alpine indentation, evidence from the Eastern Alps

**DOI:** 10.1016/j.jog.2014.07.005

**Published:** 2014-12

**Authors:** I. Bianchi, G. Bokelmann

**Affiliations:** Department of Meteorology and Geophysics, University of Vienna, Vienna, Austria

**Keywords:** Eastern Alps, Receiver functions, Moho, Anisotropy, Lower crust, Upper mantle

## Abstract

The type of collision between the European and the Adriatic plates in the easternmost Alps is one of the most interesting questions regarding the Alpine evolution. Tectonic processes such as compression, escape and uplift are interconnected and shape this area. We can understand these ongoing processes better, if we look for signs of the deformation within the Earth's deep crust of the region. By collecting records from permanent and temporary seismic networks, we assemble a receiver function dataset, and analyze it with the aim of giving new insights on the structure of the lower crust and of the shallow portion of the upper mantle, which are inaccessible to direct observation. Imaging is accomplished by performing common conversion depth stacks along three profiles that crosscut the Eastern Alpine orogen, and allow isolating features consistently persistent in the area. The study shows a moderately flat Moho underlying a seismically anisotropic middle-lower crust from the Southern Alps to the Austroalpine nappes. The spatial progression of anisotropic axes reflects the orientation of the relative motion and of the stress field detected at the surface. These observations suggest that distributed deformation is due to the effect of the Alpine indentation. In the shallow upper mantle right below the Moho interface, a further anisotropic layer is recognized, extended from the Bohemian Massif to the Northern Calcareous Alps.

## 1 Introduction

**Fig. 1:**
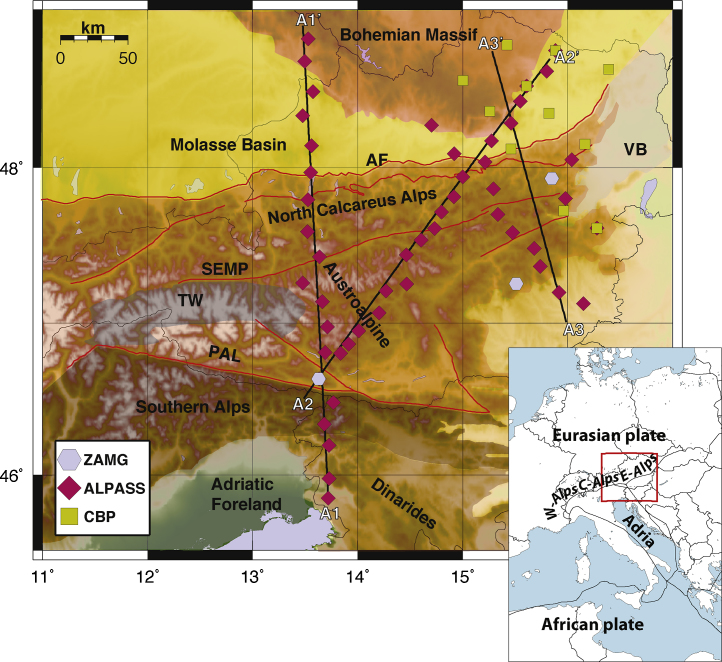
Map of the Eastern Alps with station locations. Colors are related to different networks. Red lines draw the path of major tectonic lines in the vicinity of the stations (AF = frontal Alpine thrust, PAL = Periadriatic Line, SEMP = Salzach–Ennstal–Mariazell–Puchberg fault system). Names for the different tectonic domains are written on shaded areas (TW = Tauern Window, VB = Vienna Basin). Inset shows the location of the study area in the Eastern Alps. (For interpretation of the references to color in this figure legend, the reader is referred to the web version of this article.)

The boundary between the African and Eurasian plates in the Mediterranean area consists of a broad zone of deformation, due to the convergence between the two plates (DeMets et al., 1990, DeMets et al., 1994) (Fig. 1, inset).

Since late Cretaceous the Adriatic microplate, acting as a promontory of Africa, has deeply indented Europe, resulting in yielding the Alpine orogeny (Platt et al., 1989). Within the convergence of the large (Europe and Africa) plates, the Adriatic microplate moves independently, and rotates counterclockwise with respect to stable Europe, controlling the strain pattern along its boundaries (Nocquet and Calais, 2004). The CCW rotation of Adria leads to different deformation regimes along the Alpine arc, such as: compression in the Eastern Alps, dextral shear in the Central Alps and transtension or very slow deformation in the western Alps. The shape of the eastern Alpine chain is considered to be the consequence of the tectonic activity in the Tertiary, during which contemporaneous shortening perpendicular to, and extension parallel to the orogen occurred (e.g., Ratschbacher et al., 1989).

At the surface, post-collisional shortening between the Adriatic and European plates was compensated by frontal thrusting along the frontal Alpine thrust (AF) onto the Molasse foreland basin and by contemporaneous lateral wedging of the upper plate. The main structures that bound the eastward escaping wedges are to the North the sinistral Salzach–Ennstal–Mariazell–Puchberg (SEMP) fault system, and to the South the dextral Periadriatic line (PAL) (Ratschbacher et al., 1991a, Ratschbacher et al., 1991b). The SEMP line has been recognized as a single continuous major strike-slip zone (Linzer et al., 2002), separating the North Calcareous Alps (NCA) from the eastward escaping Australpine units.

Most of the knowledge on the actual structure of the Alpine lithosphere comes from traditional body and surface wave seismic imaging which has shown high seismic velocities in the crust and upper mantle below the Alps (e.g. Lippitsch et al., 2003, Piromallo and Morelli, 2003, Diehl et al., 2009, Mitterbauer et al., 2011, Giacomuzzi et al., 2011). Deep high-velocity anomalies have been interpreted as evidence for subducted slabs, and shallower anomalies as thickened lower crust (e.g. Wortel and Spakman, 1993, Waldhauser et al., 1998, Lippitsch et al., 2003, Wagner et al., 2012). Tomographic images of the Alps have been limited to inversions for isotropic velocity structure. Anisotropic properties of the media are complementary to isotropic velocities, and provide constraints on the geodynamics of the region. Studies on seismic anisotropy in the Alps have been focusing on the larger-scale features attributed to mantle deformation of the entire Alpine region (Barruol et al., 2011, Bokelmann et al., 2013), while seismic anisotropy in the upper layers of the lithosphere has been less explored. Besides the study of Fry et al. (2010) who mapped seismic anisotropy using surface waves for the Western and Central Alps, no other study on anisotropy in the Alpine crust has been carried out so far. Zhu and Tromp (2013) describe the anisotropic properties of the lithosphere beneath Europe, and retrieve two crucial layers in which anisotropy concentrates, i.e. the lower crust and the upper mantle. The anisotropy has been interpreted as resulting from both frozen (e.g. Deschamps et al., 2008) and present sources, related to past tectonics or active crustal deformation and stress field (e.g. Crampin and Lovell, 1991). In response to strain, crustal (e.g., amphibole) and mantle (e.g., olivine) minerals can develop some specific fabrics that result in seismic anisotropy.

In the mantle, anisotropy is generally attributed to lattice-preferred orientation (LPO) of olivine minerals, and is interpreted as an indicator of strain resulting from mantle flow (e.g., Savage, 1999, Mainprice et al., 2005).

In the middle and lower crust anisotropy is a particularly important parameter for metamorphic rocks such as gneiss, amphibolite, and mica schist, in which anisotropic grade (8.3%, 9.3%, and 13.0%, respectively) is even higher than the average anisotropy in mantle dunite (8%) (Christensen and Mooney, 1995).

Tectonic processes can cause mechanical shearing, crystal reorientation, and/or re-crystallization during metamorphism that might produce textural fabrics, which are seismically anisotropic. Therefore orientation and amount of anisotropy may serve as proxies for crustal deformation (e.g. Okaya et al., 2004). Besides LPO, seismic anisotropy can be mimicked by heterogeneities at length scales smaller than the seismic waves wavelength (e.g. Hake, 1993, Fichtner et al., 2013); shape-preferred orientation (SPO) of fluid inclusions or cracks (Babuška and Cara, 1991) cause shear-wave birefringence. Finely-stratified media of different stiffness (Backus, 1962), or subhorizontal thin layers most probably have their primary origin in lithologic and metamorphic layering, including that caused by igneous intrusions and shear zones (Mooney and Meissner, 1992). These layers may often have varying amount of seismic anisotropy resulting from ductile strain.

In order to unravel the anisotropic properties of the Alpine crust and shallow upper mantle, the teleseismic receiver functions (RFs) methodology, able to detect the effect of anisotropic material at the desired scale, has been applied. The interpretation includes both radial (R) and transverse (T) components. The effects of anisotropy on the RFs dataset were illustrated in more than one theoretical study (e.g. Eckhardt and Rabbel, 2011, Nagaya et al., 2008, Levin and Park, 1998), showing the strong backazimuthal dependence of RFs on the 3D characteristics of the traversed media. This technique was applied in several places around the world with the aim of creating realistic velocity models beneath single stations or wide areas (e.g. Savage, 1998, Savage, 1999, Ozacar and Zandt, 2004, Liu and Niu, 2012, Porter et al., 2011). The use of teleseismic RFs has the advantage of not being affected by heterogeneous depth distribution of local earthquakes (as e.g. Piccinini et al., 2006, Pastori et al., 2009), since teleseismic rays sample the entire crust beneath the stations. This technique is applied for the first time in this area with the aim of defining the depth of anisotropic layers and the spatial orientation of the anisotropy symmetry axes.

## 2 Data and methods

The dataset is composed of RFs obtained from earthquakes at epicentral distance between 30° and 100°, with *M* > 5.5, selected by their high signal-to-noise ratio. Teleseismic events were recorded at 64 three-component stations, 50 belonging to the temporary ALPASS network (Mitterbauer et al., 2011), 11 to the temporary CBP network (Dando et al., 2011), and 3 stations of the permanent Austrian seismological network run by the ZAMG (Zentralanstalt für Meteorologie und Geodynamik). These stations are located between 13.5°E and 17°E longitude and between 45.5°N and 49°N latitude (Fig. 1).

The ALPASS stations were recording for one year, since July 2005 to April 2006, CBP stations from May 2006 to June 2007; from the stations belonging to the ZAMG network, we used data recorded since January 2009 to June 2011.

Seismic records are rotated into the RTZ reference system where the radial (R) is computed along the great-circle path between the epicenter and the station, positive away from the source, and the transverse (T) direction is calculated 90° clockwise from R. Receiver functions are calculated by a frequency domain algorithm using multitaper correlation estimates (see Park and Levin, 2000 for computational details). RFs are made of P-to-S (Ps) waves generated by the conversion of the incoming teleseismic P-wave into an S-wave by the passage through a seismic interface at depth (Langston, 1979, Ammon, 1991). Analysis of RF obtained at single stations of this dataset has been illustrated in Bianchi et al. (2013).

### 2.1 CCD migration

**Fig. 2:**
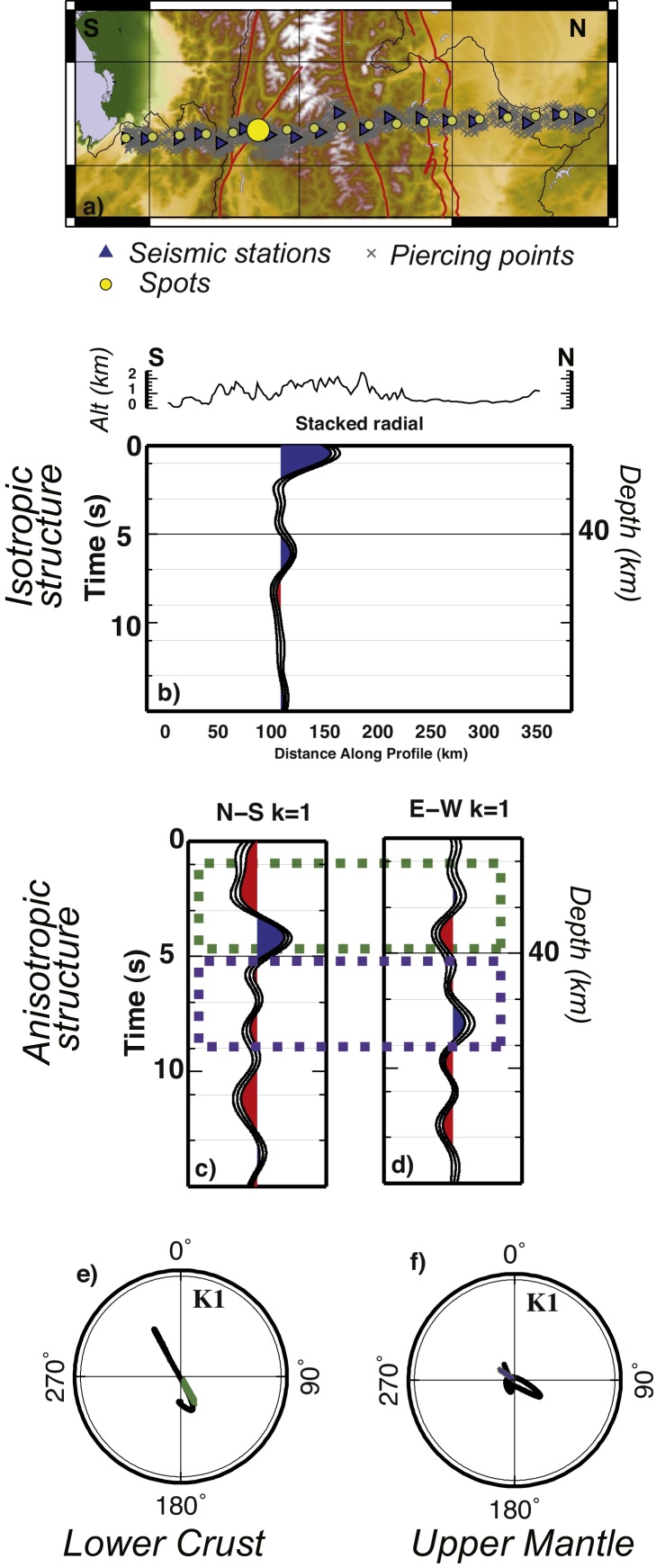
Example of RF after harmonic analysis and depth migration, and extraction of anisotropy directions. (a) Map showing the location of the seismic stations (blue triangles) of the A1-A1′ profile; gray crosses display the surface projection of the piercing points at 40 km depth for teleseismic events selected to compute the RF; yellow circles show the location of the central points (“spots”). For the 6th spot the 3 RF stacks are displayed: “stacked radial” in (b), N-S *k* = 1 in (c) and E-W *k* = 1 in (d). (e) and (f) particle motion obtained for 1–5 s and 5–9 s time-windows of the *k* = 1 traces (in (c) and (d)), employed to extract anisotropy directions in the lower crust and upper mantle. The green and violet arrows in (e) and (f) demonstrate the linear trend of the particle motion. (For interpretation of the references to color in this figure legend, the reader is referred to the web version of this article.)

To enhance the spatially continuous structures in the study area, we employ a common conversion depth technique (Dueker and Sheehan, 1998, Wilson et al., 2004) as in Bianchi et al. (2010). We select 3 main profiles across the eastern Alps (Fig. 1); profiles are labeled A1-A1′, A2-A2′ and A3-A3′ in Fig. 1, and are referred as A1, A2, A3 in the text hereinafter. For each profile, we divide the area within 40 km of the profile into rectangular boxes 20 km wide, with 50% overlapping (i.e. each area shares 50% of its surface with the adjacent areas). For each rectangular box we select the ensemble of RFs (both R and T), for which the surface projections of their conversion points at 40 km depth are inside the rectangular area, and we associate the RF stack with the center of the rectangular area (named “spot”) (Fig. 2). For our purpose, we migrate each RF at the focusing depth of 40 km, as this is the average Moho depth in the area. The velocity model used was derived from IASP91, modified to have a Moho at 40 km depth (see Table S1). The entire depth range of each trace is stacked along the move-out curve for the defined depth (i.e. 40 km). If the arrivals from a discontinuity are indeed direct Ps arrivals, then one expects the discontinuity image to come into focus when the correct depth is used (Dueker and Sheehan, 1998). For depths larger than 70 km and smaller than 20 km the distribution of conversion points of the teleseismic traces differs significantly from the one at 40 km depth, therefore to get the focus on shallower and deeper features different stacking ensembles would be needed. For each spot, we obtain an ensemble of R-RF and T-RF (location of the spots for the 3 profiles and conversion points at 40 km depth for teleseismic events used to compute the RF are illustrated in Fig. S1).

### 2.2 Harmonic analysis

Both R and T components of the RFs contain useful information on subsurface structure. The isotropic part of the seismic velocity profile at depth mainly affects the R-RF component, while anisotropy and dipping structures produce P-to-S conversion recorded on the T-RF component (Levin and Park, 1998, Savage, 1998). Here, we use a technique that allows analyzing both R- and T-RF simultaneously (Bianchi et al., 2010, Piana Agostinetti et al., 2011). This characteristic is fundamental in complex tectonic settings, e.g. subduction zones, where both anisotropy and dipping layers have been observed, together with strong isotropic seismic velocity jumps. This method is based on the extraction of the backazimuthal harmonics of a RF dataset as a function of direction of the incoming P-wavefield. The technique assumes that an ensemble of RF can be expressed as a sum of cos *kφ* and sin *kφ*, where *k* is the harmonic degree and order, and *φ* is the backazimuth. We limit our analysis to the first two degrees and orders, i.e. *k* = 0, 1. Degree and order *k* = 0 represent the bulk isotropic variation of the seismic velocities with depth (called “stacked radial” hereinafter). For flat interfaces in isotropic media, the T-RF contribution vanishes and the signals appear on the *k* = 0 backazimuthal harmonic (i.e. a simple stack of the R-RF components). The first harmonic, *k* = 1 contains the energy which displays a periodicity of 2*π* with the backazimuth *φ*. The 2-lobed periodicity is expected for a dipping interface or an anisotropic layer with a plunging symmetry axis at depth (Maupin and Park, 2007). In such cases, the R- and T-RF backazimuthal harmonics display amplitude variability with backazimuth *φ*, with a *π*/2 shift (Shiomi and Park, 2008). So, for *k* = 1, we sum the R- and T-RF backazimuthal harmonic with a phase shift of +*π*/2 to enhance the effect of anisotropy or dipping interfaces. Details on the harmonic decomposition are given in the Appendix and in Bianchi et al. (2010).

In the presence of an interface dipping toward North or South, or in case of an anisotropic symmetry axis trending North or South, the cos 1*φ* (i.e. *k* = 1) back-azimuthal harmonic has maximum absolute amplitudes. In case of E-W oriented structures, i.e. interfaces dipping down to the East or down to the West, and symmetry axes East or West trending, the sin 1*φ* (i.e. *k* = 1) back-azimuth harmonic has the largest absolute value. Thus we name the term cos *φ* and sin *φ* as N-S (*k* = 1) and E-W (*k* = 1) terms, respectively.

Although the signal of a dipping layer in a RF dataset (i.e. a layer bounded by two parallel dipping interfaces) and a layer with plunging anisotropic axis generate phases on R and T with the same periodicity (see Sherrington et al., 2004, Bianchi et al., 2008, Bianchi et al., 2010) and therefore appear in the same fashion on the N-S (*k* = 1) and E-W (*k* = 1) gathers of the dataset, here we interpret signals as due to anisotropy. Matching observed amplitudes with dipping layers typically requires unreasonably steep dips (Leidig and Zandt, 2003, Savage, 1998), while we do not observe depth variation of the pulses along profiles and among profiles as well, facing rather subhorizontal structures.

The second harmonic, *k* = 2 contains the energy which displays a *π*-periodicity (4-lobed) with backazimuth *φ* of the incoming P wave; this occurs for subsurface structures including an anisotropic layer with horizontal symmetry axis (Maupin and Park, 2007). No coherent signal is observed on these components; therefore they are not included into this analysis. Fig. S2 in the electronic supplementary material shows the *k* = 2 components for each profile together with the backazimuthal distribution of events used to build the traces.

### 2.3 Anisotropy

Fig. 2 shows the three traces obtained for one RF ensemble. The incoming teleseismic P-wave is partly converted to SV- and SH-wave when hitting the interface of an anisotropic layer. The trend of an anisotropy symmetry axis (*Φ*) can be estimated from the phases observed on the N-S (*k* = 1) and E-W (*k* = 1) components of the harmonic analysis. The time delay between direct and converted phase is related to the conversion depth. Depth and *Φ* are estimated by isolating phases within a specified time window. Our first means of estimating *Φ* is thus based on the ratio of peak energy in the harmonic terms for the selected time window.

The 2 terms of the first order harmonic (*k* = 1) are thus used to constrain the trend of the anisotropic axis. According to the construction of the harmonic components, the first term is maximum for a N-S oriented symmetry axis, while the second one is maximized for an E-W oriented symmetry axis. The ratio thus gives the azimuth of the symmetry axis, and the polarity of the pulses indicates the trend; N and E trending fast symmetry axes generate an earlier pulse of positive polarity (blue) while S and W trending fast symmetry axes generate an earlier pulse of negative polarity (red) (see Bianchi et al., 2010). The symmetry axis may be associated with fast (positive) or slow (negative) velocity. These can generate similar effects in the data when the two have opposite trend (*Φ* 180°) and complementary (90°–*δ*) plunge (Sherrington et al., 2004 and references therein). To reproduce the same pattern, slow symmetry axes should therefore trend in the opposite direction (Sherrington et al., 2004, Bianchi et al., 2008, Bianchi et al., 2010). Hypothesized anisotropy is modeled using hexagonal symmetry, with a unique fast or slow symmetry axis and uniformly slow or fast velocities, respectively, for seismic wave propagation directions in the plane perpendicular to that axis (Weiss et al., 1999). Whereas other symmetries can be present in the Earth, this is the simplest geometry able to reproduce the major observable effects. The assumption of hexagonal symmetry is appropriate for the mantle (Becker et al., 2006), as well as the crust (Sherrington et al., 2004, Ozacar and Zandt, 2004, Eckhardt and Rabbel, 2011). A number of geologically feasible scenarios could result in relatively uniform seismic anisotropy at large scale within the crust, among which are aligned microcracks, and alignment of mineral grains in a large body of rock (Rabbel and Mooney, 1996). Uniform seismic anisotropy at large (teleseismic) scale could be present in several geological settings. While mantle anisotropy is widely accepted to be due to physical properties of the aligned olivine crystals, there are many potential causes for crustal anisotropy. Among these, the main factors are the presence of non-hydrostatic stress inducing aligned micro-cracks in the shallow crust, the layering of sedimentary piles, and the alignment of anisotropic mineral grains in deeper portion of the crust, i.e., lamination of the lower crust (Meissner et al., 2006). All these causes can be approximated using hexagonally anisotropy with a unique slow or fast symmetry axis, respectively. Discriminating between the two kinds of anisotropy could be a challenge, and available geological information is needed to give a priori constrains. In this study we consider a fast symmetry axis for both the middle-lower crust and upper mantle. Due to the lack of information on the slow or fast nature of anisotropy in the lower crust, and since the two different symmetry axes (fast and slow) generate the same feature on RF if trending with an opposite angle and plunging of a complementary angle, the choice of one or the other is arbitrary.

We employ the linear fit to the particle motion of the two *k* = 1 terms (in the defined time window, i.e. 1–5 s for the crust and 5–9 s for the mantle) in order to indicate the orientation of the fast anisotropic axis, while we employ the polarity of the pulses to indicate the trend of the axis (Fig. 2c–f). This is only possible when top and bottom of the anisotropic layer are identified, otherwise, if one of the contrasts at the top or at the bottom of the anisotropic layer is gradual, not producing a conversion, it would not be possible to identify the anisotropic layer. Both, plunge of the symmetry axis as well as strength of anisotropy are dependent on the phase amplitude (e.g. Ammon et al., 1990). The tradeoff between the two can be addressed by waveform modeling. In this study we use the pulse amplitude as a proxy to indicate the relative strength of anisotropy, since a variation of the plunge of the anisotropy axis, instead, would imply a change of the deformation regime along the profiles. For this reason, the most robust result of our work is the trend of the symmetry axes, and we will discuss this only.

The particle motion, from which we retrieve the trend of anisotropy axes, has been calculated for all stations between two characteristic time windows: 1–5 s (in the crust) and between 5 and 9 s (in the subcrustal mantle) (as shown in Fig. 2c and d).

## 3 Results

In the following we will present the most robust features of the dataset. The simplest way to test the robustness of one feature with respect to others is its spatial continuity through the dataset. This approach constraints us to larger-scale features emerging from the observations. Two main features are going to be discussed: (1) the occurrence of the Moho phase; (2) the occurrence of phases related to anisotropic layers above and below the Moho.

### 3.1 Moho

**Fig. 3:**
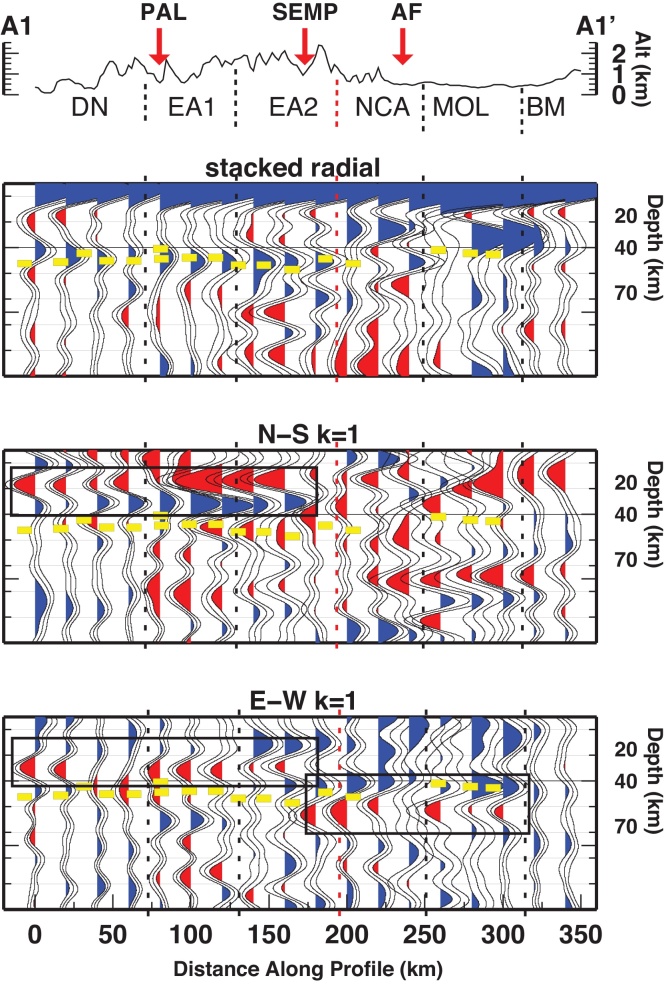
RF dataset along profile A1-A1′, displayed as stacked radial, N-S and E-W backazimuthal harmonics. Blue and red pulses indicate positive and negative amplitudes, respectively. Yellow dashes mark the Moho depth from Bianchi et al. (2013). Black rectangles highlight pulses described in the text. Vertical dashed lines divide the profile according to similar features. (For interpretation of the references to color in this figure legend, the reader is referred to the web version of this article.)

**Fig. 4:**
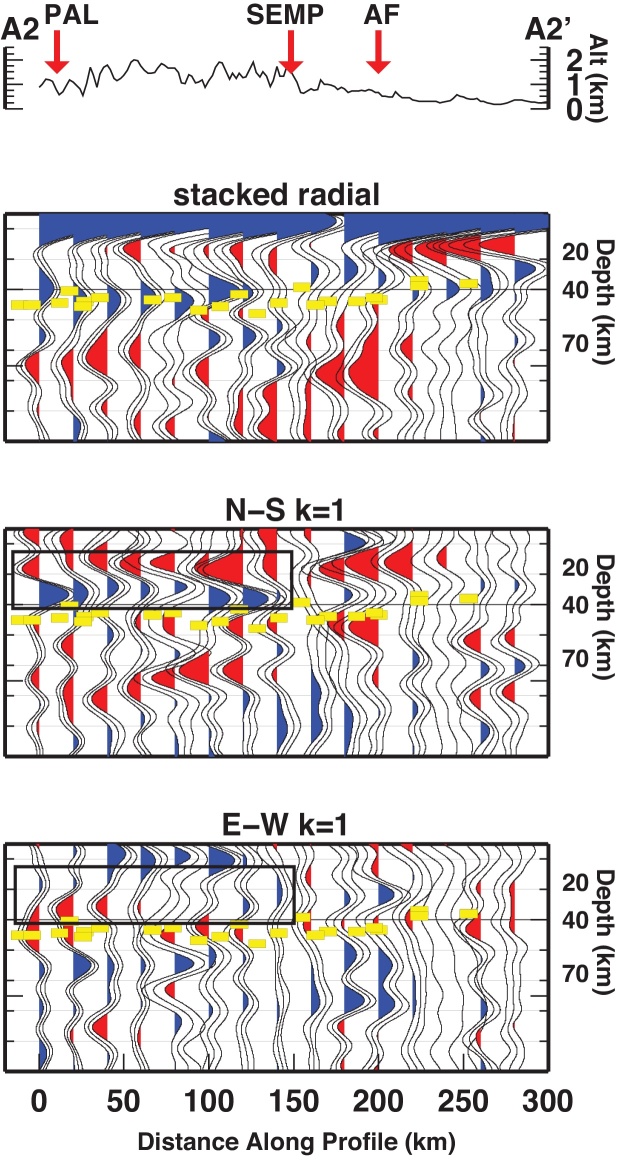
Same as Fig. 3 for profile A2-A2′.

**Fig. 5:**
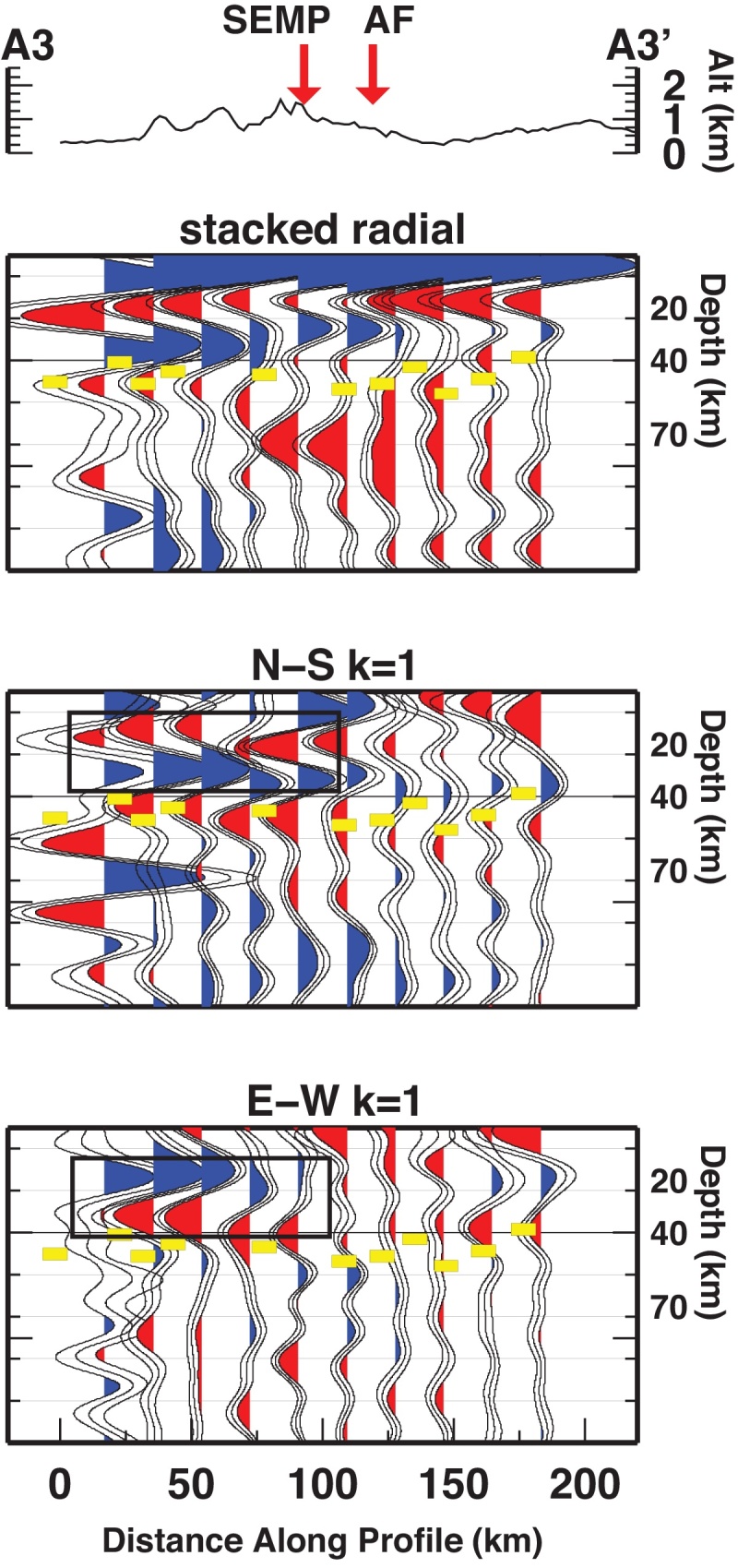
Same as Fig. 3 for profile A3-A3′.

The crust-mantle boundary (Moho) is the most pronounced seismic contrast in the lithosphere and therefore, the Ps phases converted at the Moho dominate the RF time series. The prominent phase observed on the stacked radial gather is therefore associated with the Moho interface. Moho depth from RF at single stations retrieved by Bianchi et al. (2013) are projected along the three profiles (yellow dashes in Fig. 3, Fig. 4, Fig. 5). Moho depth variations are observed along the profiles. They witness a deeper interface to the South and a shallower interface to the North. The transition between deep and shallow Moho occurs between the SEMP and AF fault lines (profiles A1 and A2). Whether the deep-to-shallow transition is abrupt or smooth, or if the two interfaces are overlapping is not yet detectable with this analysis and by the employed frequencies.

In the northern portion of the A1 profile, at 300–360 km distance, two close interfaces occur, the Ps indeed is composed of two merged Ps phases resulting in a broad pulse. At 210–300 km the two Ps phases are separated, the related interfaces would occur at about 30 and 50 km depth. The extension of these features along the profile is not clear, and an area of crustal doubling between Adriatic and European crusts is possible. In the southern part of profile A1, a sharp phase between 0 and 210 km along profile is recognized, due to an interface located between 40 and 50 km depth.

Profile A2 features a deep-to-shallow Moho transition between 120 and 160 km. In the southern portion of A2 (0–120 km), the phase related to the Moho occurs between 40 and 50 km depth. In the northern part of A2 (160–300 km), the Moho phase occurs between 30 and 40 km depth.

Along profile A3 the transition between deep and shallow Moho appears smooth, but the occurrence of the Moho phase is in disagreement with the projected Moho depth retrieved by single station analysis, being at 40 km depth in the southern edge of the profile, and at about 25 km depth at the northern edge.

### 3.2 Anisotropy

*k* = 1 components of the harmonic analysis have been employed to extract the azimuth and the dip direction of the symmetry axes. We focus here on the features outlined in Fig. 3, Fig. 4, Fig. 5 along the three profiles, where we observe two consecutive pulses of opposite polarity (coupled pulses) at earlier arrival time than the Moho pulse (corresponding to depths between 20 and 40 km), and coupled pulses at arrival times later than the Moho pulse (depths between 40 and 60 km).

Symmetry axis azimuths were extracted by the particle motion derived from the selected time windows in the *k* = 1 components, while dip directions are detected by the polarity.

**Fig. 6:**
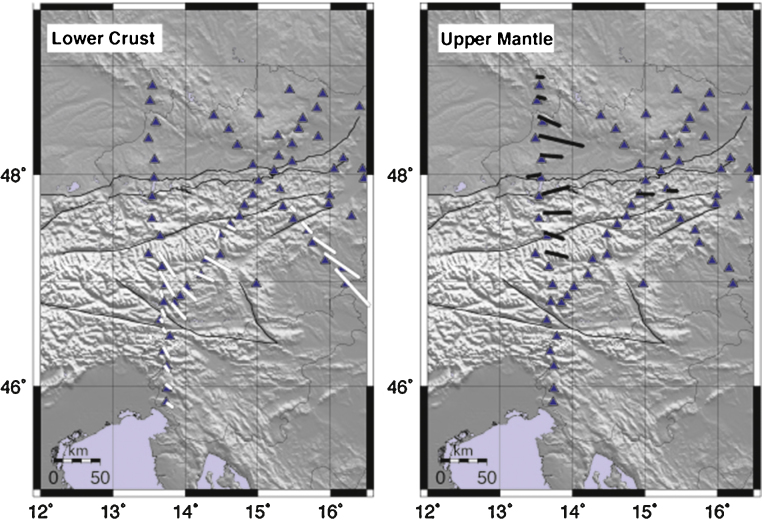
Maps of anisotropy directions detected in the lower crust and upper mantle. Black thin lines represent the major tectonic lineaments.

Profile A1 shows two strong pulses (along the N-S (*k* = 1) component) coherent within 0–160 km along profile. Their arrival time is earlier than the deep Moho pulse observed in the stacked radial at the southern half of the profile. This feature abruptly ends at 160 km distance along the profile. Its northward extension corresponds to the northward termination of the deep Moho phase. This feature is also associated with a coupled pulse on the E-W (*k* = 1) component. The polarity of the phases is constant for the southern 160 km, while amplitudes are increasing toward the North (from 60 to 160 km the traces display larger amplitudes than those between 0 and 60 km). Particle motions indicate a SE-trending symmetry axis, with increasing length toward the North (Fig. 6).

Profile A2 and A3 feature the same phases, for the same time windows, respectively within 0–140 km and 0–90 km along the profile.

A second stable coupled pulse is observed at arrival times larger than the Moho phase along profile A1 on the E-W *k* = 1 component within 180–300 km. A similar phase is rarely visible along other profiles. Particle motions indicate an E-trending symmetry axis (Fig. 6).

### 3.3 Synthetic test

**Fig. 7:**
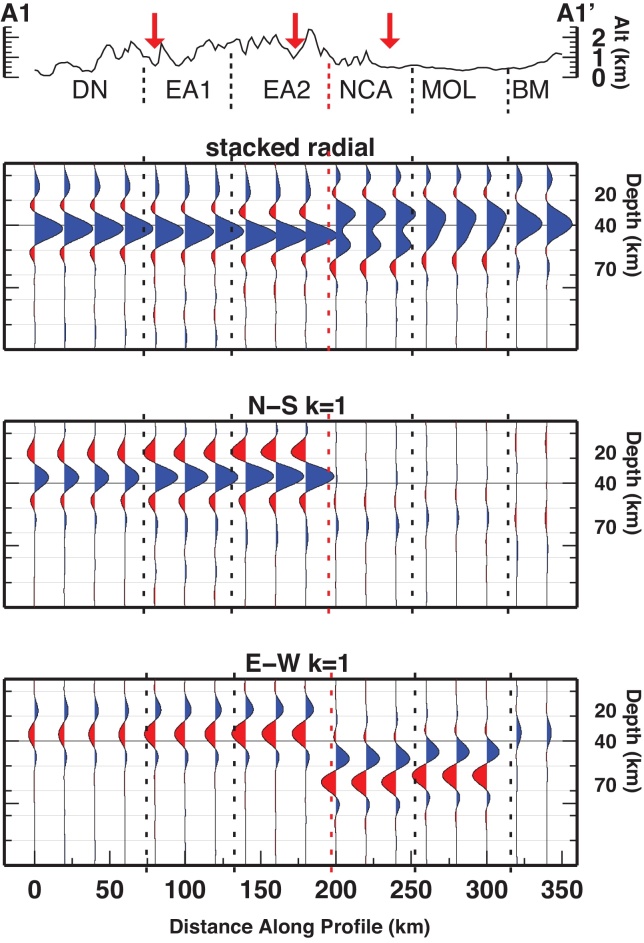
RF synthetic dataset displayed as stacked radial, N-S and E-W backazimuthal harmonics. Vertical dashed lines divide the profile according to different models used to compute the RF, the division follows the observations in Fig. 3. Velocity models employed to obtain the synthetics are described in Table S2.

A simple synthetic test reproducing the main features of profile A1 is used to confirm the occurrence of anisotropy in the two distinct layers of the lithosphere (i.e. mid-lower crust and upper mantle) and to sustain the interpretation of the features encountered in the RF dataset. The synthetic RF dataset (Fig. 7) is created using RAYSUM (Frederiksen and Bostock, 2000) and is compared to the observed receiver functions along profile A1 (Fig. 3). The S-velocity models employed for the synthetics computations have been constructed to match the observations on phase arrival times and polarity along profile A1, and are resumed in Table S2.

The profile has been divided into two domains (southern and northern). The boundary between southern and northern domain is geographically located between the SEMP fault and the AF fault. Each domain is then split into 3 subdomains according to similarities in the observed traces (along profile A1). The southern domain features a dipping converter (Moho) at 40–45 km depth (South toward North). In the crust between 15 and 35 km depth an anisotropic layer with symmetry axis trending N155° matches the observed *k *= 1 components. The difference among the three southern subdomains lies in the stronger anisotropy from South to North (5% in the foreland (i.e. DN in Fig. 7), 8% in the southern part of the Southern Alps (i.e. E-Alp1 in Fig. 7) and 10% in the northern part of the southern domain (i.e. E-Alp2 in Fig. 7)).

The northern domain has been modeled with two interfaces at depth: a flat interface at 30 km depth, and a dipping interface at a depth of 40 km below the Bohemian Massif, going down to 50 km depth below the Northern Calcareous Alps nappe. Below that dipping interface an anisotropic layer is placed with 4% anisotropy with an axis trending N100°.

## 4 Discussion

The transition from the crust to the mantle, the Moho, is one of the main features appearing in receiver functions, and we observe it in these data. The frequency content of the teleseismic records is low. Therefore this technique is not able to resolve the fine structure of the Moho, although it can provide relatively reliable maps of its lateral variation at larger scale. As Fig. 2a shows, the station spacing is responsible for the lateral resolution of the image, while the number of teleseismic records (redundancy) acquired at each station is responsible for the robustness of the observations.

A positive phase (increase in velocity with depth) has been detected along the three profiles and interpreted as the Moho. Delay times between the direct P and Ps-converted phases at the Moho are larger for the southern parts of the three profiles and smaller for the northern parts. The transition between these deep and shallow Moho depths occurs between the SEMP and AF fault lines for profiles A1 and A2. If the deep-shallow transition corresponds to the plate boundary, this would be located about 100 km to the North of the surface location of the PAL where the plate boundary has been interpreted to be by Brückl et al. (2010).

**Fig. 8:**
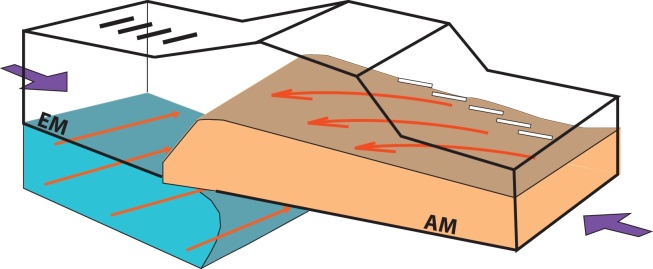
Synthetic sketch summarizing the relation of the anisotropic bodies detected in this study and the geodynamic processes in the area. The anisotropic lower crust (orange area) is located in the southern region and the anisotropic upper mantle (blue area) in the northern region. Violet arrows show the N-S-directed compression, red arrows for the eastward extrusion of the EA in the mantle, and counterclockwise rotation of Adria in the middle-lower crust. Black and white sticks show the orientation of the anisotropic axes as in Fig. 6. EM = European Moho; AM = Adriatic Moho. (For interpretation of the references to color in this figure legend, the reader is referred to the web version of this article.)

Consistent features observed along profiles suggest the occurrence of anisotropic layers in the mid-lower crust and upper mantle. Crustal rocks can be extremely anisotropic (e.g. Christensen, 1966) and there is a close relationship between observed seismic anisotropy and rock structure, e.g. foliation and lineation of deeply-buried rocks (Barruol and Kern, 1996), which are otherwise not directly accessible. Observations of seismic anisotropy within the upper mantle and middle-lower crust may be fundamental to the understanding of plate and intra-plate tectonics (Owens and Bamford, 1976). Therefore the following discussions focus on outlining the relation between tectonic history of the study area and the retrieved orientations of seismic anisotropy. Fig. 8 schematically illustrates the relation between anisotropic bodies at depth and the geodynamic processes in the area.

The major mechanisms that influence tectonic evolution of the area are the N-S contraction caused by the collision of Europe and Adria, the counterclockwise rotation of the Adriatic microplate, and the Eastward extrusion toward the Pannonian Basin. The lateral (east-directed) extrusion occurred after the initial contraction, as an escape of the lithospheric wedge toward the space left from the Carpathians rollback and tectonic relaxation.

Geodetic data generally agree that the Adriatic indenter, as an independent microplate detached from the African plate, rotates counterclockwise with respect to stable Europe (Anderson and Jackson, 1987, Westaway, 1990, Calais et al., 2002). This suggests that active deformation in the Alps is controlled, and possibly driven, by the motion of the Adriatic microplate. The location of profile A1 coincides with the area subject to NNW-oriented motion (from GPS) with respect to Eurasia (Devoti et al., 2008). The same area displays NNW-SSE orientation of the stress field (Heidbach et al., 2008).

Therefore the SE and SSE detected trend of anisotropy, reflects the orientation of relative motion across the Alps and of the stress field at the surface, suggesting that associated deformation is occurring in the middle-lower crust of the South-Alpine region. This observation is comparable with azimuthal anisotropy SSE oriented within a depth of 50 km detected in the Southern Alpine area by Zhu and Tromp (2013).

A wedge of Adriatic lower crust is described in Rosenberg and Kissling (2013) 3.5° to the West with respect to our A1 profile, terminating below the northern margin of the Engadine window that lies at the same latitude of the northward extension of the anisotropy observed in this study. The anisotropic Adriatic middle-lower crust wedges toward the North and approximately ends at the latitude of the SEMP fault. Interestingly, this feature is visible to the South and the North of the PAL, which may suggest that the deformation has occurred (or is occurring) later than the times when the PAL was active, or that the extension of the PAL is limited to the upper crust. As described above, the area is currently active with NNW-SSE compression, a direction which also shows in earthquake focal mechanisms (e.g., Cheloni et al., 2012). The style of deformation in the deep crust to both sides of the fault is similar, suggestion that the viscosity of the middle-lower crust is not very different.

E-trending anisotropy is identified in the northern part of the profile, at a depth larger than 40 km. At these depths the rocks constituting the mantle behave in an increasingly ductile manner and record the latest deformations. If the mantle flows toward the East with a larger velocity with respect to the crust, then the olivine symmetry axes undergo alignment (for simple shear), resulting in an EW-oriented fast anisotropy axis with a dip toward the East (e.g., Bokelmann, 2002).

This corresponds to fast anisotropy orientations from SKS splitting and adjoint tomography that show strong correlation with the arcuate shape of Alpine topography (Bokelmann et al., 2013, Zhu and Tromp, 2013) being E-W and ESE oriented in this area. Both SKS and the seismic tomography sample larger structures compared to our study. Nevertheless orientations of the results from the different methodologies are comparable. Under our stations the deformation giving rise to EW-oriented symmetry axes may perhaps act at deeper layers, and the crustal signal may also obliterate the deeper signal from the mantle at the employed frequencies.

## 5 Conclusions

This study presents the results of a receiver function analysis on a densely spaced seismic network in the Eastern Alps. The applied method is based on the harmonic decomposition of the receiver functions exploiting information contained in both radial and transverse components of the data. The technique is used to retrieve the isotropic structure under the seismic stations and to focus on anisotropic properties at two critical depths: (1) the middle and lower crust and (2) the uppermost mantle. In the middle-lower crust the anisotropy is concentrated in the southern part of the study region. It shows a spatially consistent pattern with anisotropic symmetry axes dipping to the SSE, in a direction that is not unlike the orientation of the geodetic motion and of the stress field detected at the surface, which are elements of the North–South convergence and the counterclockwise rotation of the Adriatic microplate.

In the uppermost lithospheric mantle, there is an anisotropic signal in the northern part of the study area, where eastward trending symmetry axes are observed. This is in rough agreement with fast orientations from the recent SKS-splitting study of Bokelmann et al. (2013).

## Appendix C [{(Appendix)}]

Linear regression of the R-RF and T-RF is computed as follows: given *n* couples of R- and T-RF, for each time *t*, the coefficients of the cos k*φ* and sin k*φ*, with *k* = 0, 1, 2, terms are computed solving this linear system:

$$\left(\begin{array}{c} R_{1}(t)\\ ...\\ R_{n}(t)\\ T_{1}(t)\\ ...\\ T_{n}(t) \end{array}\right)=\left(\begin{array}{ccccc} 1 & \cos\;\varphi_{1} & \sin\;\varphi_{1} & \cos\;2\varphi_{1} & \sin\;2\varphi_{1}\\ ... & \; & \; & \; & \;\\ 1 & \cos\;\varphi_{n} & \sin\;\varphi_{n} & \cos\;2\varphi_{n} & \sin\;\varphi_{n}\\ 0 & \cos\;\varphi_{1}+\pi /2 & \sin\;\varphi_{1}+\pi /2 & \cos\;2\varphi_{1}+\pi /4 & \sin\;2\varphi_{1}+\pi /4\\ ... & \; & \; & \; & \;\\ 0 & \cos\;\varphi_{n}+\pi /2 & \sin\;\varphi_{n}+\pi /2 & \cos\;2\varphi_{n}+\pi /4 & \sin\;2\varphi_{n}+\pi /4 \end{array}\right)\left(\begin{array}{c} A(t)\\ B(t)\\ C(t)\\ D(t)\\ E(t) \end{array}\right)$$

*A*(t) represents the constant part of the RF dataset, while *B*(t) and *C*(t) are the amplitudes of the cos *φ* and sin *φ* terms (also called *k* = 1 terms) analyzed in this study. The coefficients *D*(t) and *E*(t) are the amplitudes of the *k* = 2 terms, shown in Fig. S2 of the electronic supplement for each profile.
