# Supplementary material for: Seismic signature of the Alpine indentation, evidence from the Eastern Alps
Source: J Geodyn. 2014 Dec;82:69–77. doi: 10.1016/j.jog.2014.07.005 (PMC4599446; doi:10.1016/j.jog.2014.07.005)
Supplement: Supplementary file 1 [file mmc1.docx]

Table S1. Velocity models used to depth convert the RFs. In the columns from left to the right: depth range (km); P-wave velocity (km/s); S-wave velocity (km/s), density (kg/m^3^)

Table S2. Velocity models used to construct synthetic receiver functions shown in Figure 7. The velocity models refer to the portions of the profile displaying the same abbreviation as in brackets. In the columns from left to the right: depth range (km); S-wave velocity (km/s); anisotropy %; azimuth of the anisotropy axis (° from N towards E); anisotropy inclination (° from horizontal).

Figure S1. Maps displaying the locations of the 3 profiles, with dots representing the spots for which we estimated the RF. Grey crosses for piercing points at 40 km depth for events used to construct each profile.

Figure S2: Panels a,c,e: cos 2ϕ components of the harmonics analysis for profiles A1, A2 and A3 respectively. Panels b,d,f: sin 2ϕ components of the harmonics analysis for profiles A1, A2 and A3 respectively. g) Backazimuthal distribution of the events used to build the RF stack for spot 13 in profile A1; this trace counts the smallest number of events used for a stack construction. h) Backazimuthal distribution of the events used to build the RF stack for spot 7 in profile A3; this trace counts the largest number of events used for a stack construction.
