# Supplementary material for: Seismic signature of the Alpine indentation, evidence from the Eastern Alps
Source: J Geodyn. 2014 Dec;82:69–77. doi: 10.1016/j.jog.2014.07.005 (PMC4599446; doi:10.1016/j.jog.2014.07.005)
Supplement: Supplementary file 2 [file mmc2.docx]

Table S1

| *Depth range (km)* | *Vp (km/s)* | *Vs (km/s)* | *Density*  *(kg/m^3^)* |
| --- | --- | --- | --- |
| *0-20* | *5.8* | *3.36* | *2.7* |
| *20-30* | *6.5* | *3.75* | *2.7* |
| *30-40* | *6.6* | *3.8* | *3.0* |
| *halfspace* | *8.04* | *4.47* | *3.3* |
