# Supplementary material for: Seismic signature of the Alpine indentation, evidence from the Eastern Alps
Source: J Geodyn. 2014 Dec;82:69–77. doi: 10.1016/j.jog.2014.07.005 (PMC4599446; doi:10.1016/j.jog.2014.07.005)
Supplement: Supplementary file 3 [file mmc3.docx]

Table S2. Velocity Models

| *Depth range (km)* | *Vs (km/s)* | *Anisotropy %* | *Anisotropy azimuth (°)* | *Anisotropy*  *inclination (°)* |
| --- | --- | --- | --- | --- |
| *Dinarides (DN)* | | | | |
| *0-15* | *3.5* | *-* | *-* | *-* |
| *15-35* | *3.65* | *5* | *155* | *30* |
| *35-40* | *3.65* | *-* | *-* | *-* |
| *halfspace* | *4.4* | *-* | *-* | *-* |
| *Eastern Alps1 (EA1)* | | | | |
| *0-15* | *3.5* | *-* | *-* | *-* |
| *15-35* | *3.65* | *8* | *155* | *30* |
| *35-42* | *3.65* | *-* | *-* | *-* |
| *halfspace* | *4.4* | *-* | *-* | *-* |
| *Eastern Alps2 (EA2)* | | | | |
| *0-15* | *3.5* | *-* | *-* | *-* |
| *15-35* | *3.65* | *10* | *155* | *30* |
| *35-45* | *3.65* | *-* | *-* | *-* |
| *halfspace* | *4.4* | *-* | *-* | *-* |
| *North Calcareous Alps (NCA)* | | | | |
| *0-30* | *3.5* | *-* | *-* | *-* |
| *30-50* | *4.2* | *-* | *-* | *-* |
| *50-70* | *4.2* | *4* | *100* | *20* |
| *halfspace* | *4.5* | *-* | *-* | *-* |
| *Molasse (MOL)* | | | | |
| *0-30* | *3.5* | *-* | *-* | *-* |
| *30-45* | *4.2* | *-* | *-* | *-* |
| *45-65* | *4.2* | *4* | *100* | *20* |
| *halfspace* | *4.5* | *-* | *-* | *-* |
| *Bohemian Massif (BM)* | | | | |
| *0-30* | *3.5* | *-* | *-* | *-* |
| *30-40* | *4.0* | *-* | *-* | *-* |
| *halfspace* | *4.5* | *-* | *-* | *-* |
