# Supplementary figures and images for: Seismic signature of the Alpine indentation, evidence from the Eastern Alps
Source: J Geodyn. 2014 Dec;82:69–77. doi: 10.1016/j.jog.2014.07.005 (PMC4599446; doi:10.1016/j.jog.2014.07.005)

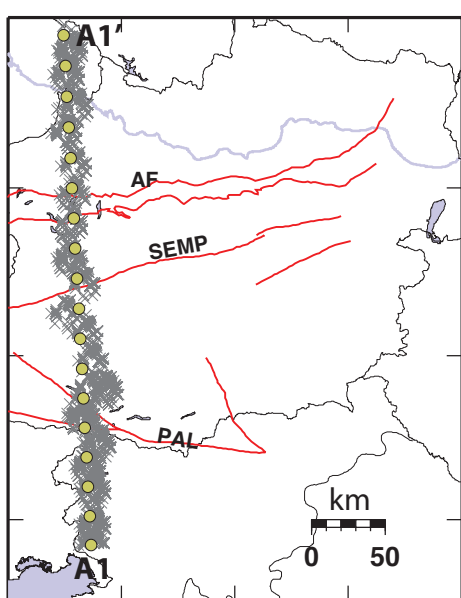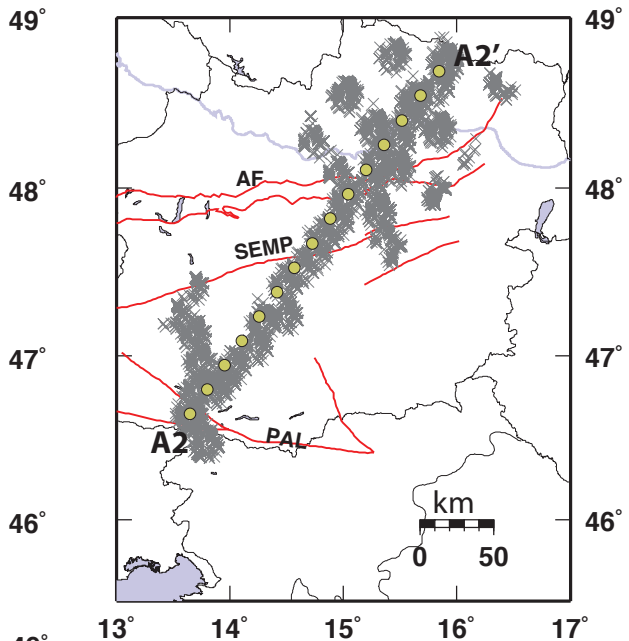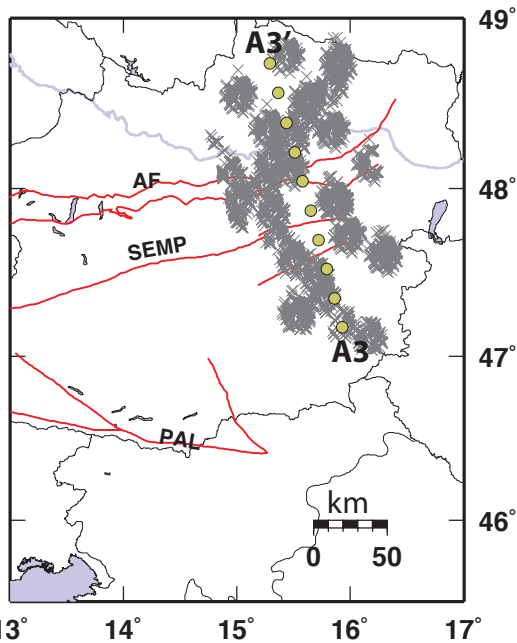

Supplement: Supplementary file 4 [file mmc4.pdf]

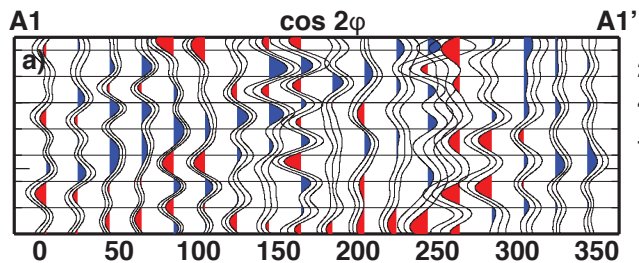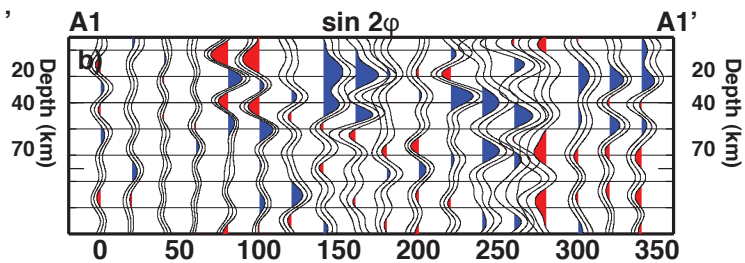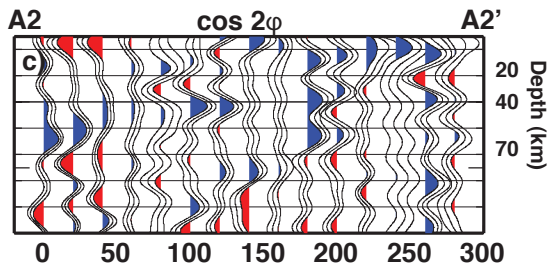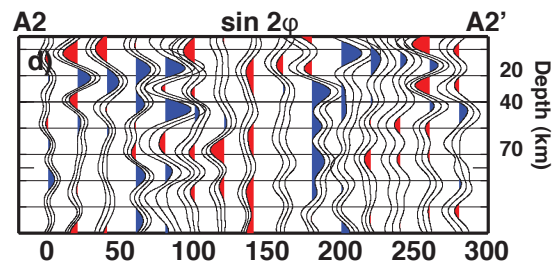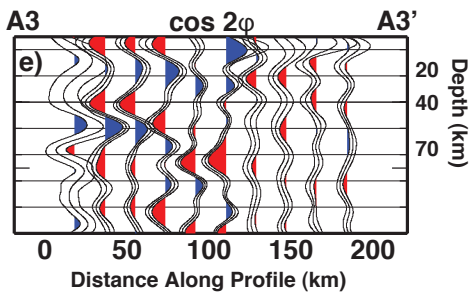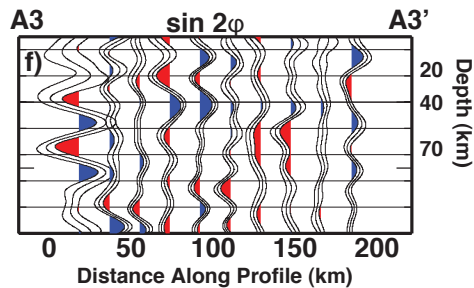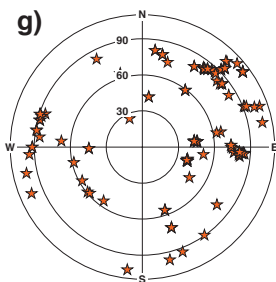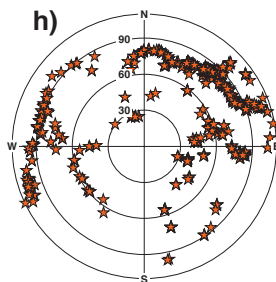

Supplement: Supplementary file 5 [file mmc5.pdf]
